# Factors Influencing Older Adults’ Perception of the Age-Friendliness of Their Environment and the Impact of Loneliness, Technology Use, and Mobility: Quantitative Analysis

**DOI:** 10.2196/67242

**Published:** 2025-05-06

**Authors:** Eric Balki, Niall Hayes, Carol Holland

**Affiliations:** 1 Department of Health Research Faculty of Health and Medicine Lancaster University Lancaster United Kingdom; 2 Keyworth Building Leeds University Leeds United Kingdom

**Keywords:** COVID-19, age-friendliness of environments, physical isolation, digital communication technologies, loneliness, cross-sectional, WHO, World Health Organization, older adults, reduced mobility, age friendliness of environments, adult well-being, social connections, aging in place, life-space mobility, LSE, functional mobility, UCLA loneliness scale, age-friendly environment assessment tool, AFEAT

## Abstract

**Background:**

The World Health Organization’s (WHO) publication on age-friendly environments (AFEs) imagines future cities to become more age-friendly to harness the latent potential of older adults, especially those who have restricted mobility. AFE has important implications for older adults in maintaining social connections, independence, and successful aging-in-place. However, technology is notably absent in the 8 intersecting domains of AFEs that the WHO imagines improve older adult well-being, and we investigated whether technology should form a ninth domain. While mobility was severely restricted, the COVID-19 pandemic provided an opportunity to test how older adults’ perceptions of their AFE changed and what role technology was playing.

**Objective:**

This study examined how life-space mobility (LSM), a concept for assessing patterns of functional mobility over time, and loneliness impacted perceived AFEs and the moderating effect of technology. It also explores whether technology should play a greater role as the ninth domain of the WHO’s imagination of the AFE of the future.

**Methods:**

In this cross-sectional quantitative observation study, data from 92 older adults aged 65-89 years were collected in England from March 2020 to June 2021 during the COVID-19 pandemic. The Life-space Questionnaire, Technology Experience Questionnaire, UCLA (University of California, Los Angeles) Loneliness Scale, and age-friendly environment assessment tool were used. Correlation and moderation analyses were used to investigate relationships between variables.

**Results:**

Most participants (86/92, 93%) had not left their immediate town in the previous 4 weeks before the interview. Restricted LSM was positively correlated to the age-friendly environment assessment tool, that is, rising physical isolation was linked to a better perception of AFEs; however, we discovered this result was due to the moderating impact of increased use of technology, and that restricted LSM actually had a negative effect on AFEs. Loneliness was correlated negatively with the perception of AFEs, but technology use was found to moderate the impact of loneliness.

**Conclusions:**

Pandemic-related LSM restrictions impacted perceived AFEs and loneliness negatively, but technology played a moderating role. The findings demonstrate that technology could be considered as a ninth domain in the WHO’s assessment of AFEs for older adults and that there is a need for its explicit acknowledgment.

## Introduction

### Background

Enabling the development of age-friendly environments (AFEs), defined as a physical and social setting that helps people age well and participate in their communities to promote “aging-in-place” for the mental and physical well-being of older adults, has become an increasingly important policy issue. This is in part a response to an aging population dynamic, urbanization, intensification of housing concerns, and community preferences, causing an increase in the deterioration of mental wellness, anxiety, stress, and depression among other disorders [1]. Older adults’ perception of AFEs directly affects their quality of life (QOL) and known predictors of depression, including loneliness [2,3]. In urban environments, older adults tend to spend much of their time in their local neighborhoods and are therefore sensitive to change [4,5]. Environmental degradation, such as lack of resources, restricted access to health care, or crime, brings additional challenges.

Previous studies have demonstrated that the perception of AFEs affects the action space of older people, affecting their social participation [6]. It is known to positively moderate the relationship between frailty, mental well-being, and depression, particularly in older adults with poor health, limited mobility, and cognitive decline [1,7,8]. Moreover, a lack of social opportunities can result in loneliness, social isolation, and worsened depression, all of which exacerbate cognitive decline, QOL, and frailty [8-14]. Aboderin et al [15] and Ng et al [16] also noted a reduction in older adults’ psychological resilience due to sustained stress and anxiety resulting from crowding, lack of space, and other issues that revolve around a poor age-centered environment. Other research has demonstrated a correlation between loneliness and AFEs [17-21], and identified factors linking the psychological health of economically disadvantaged older adults with their perception of AFE. Rantakokko et al [22] described the impact of person-environment interaction on mental well-being, demonstrating a mechanism by which older adults who experienced loneliness perceived obstacles with their environment. Stafford [23] demonstrated that older age insularity and the accompanying withdrawal from social interaction can result in deteriorating relationships, loneliness, poorer mental health, and a diminished perception of AFEs. If the environment is not conducive to aging-in-place, it causes difficulties in accessing services [24] and an increased risk of physical and mental health care needs [25]. As such, the perception of AFEs must be considered carefully to improve understanding of older adults aging-in-place and mental well-being.

Life-space mobility (LSM) describes the physical environment a person inhabits daily, structured into various zones (called life-space zones), centered on an anchor (eg, bedroom), and expanding outward into the rest of the house, house perimeter, local community, neighborhood, or town [26,27]. This concept corresponds to individuals’ functional mobility enabling meaningful participation in community activities. It reflects how older adults move across life-space zones over a given period while incorporating frequency and independence.

According to the United Nations Development Program report “The Sustainable Development Goals and COVID-19” [28], there are unprecedented ongoing burdens on mental health for older adults. Social distancing orders during the COVID-19 pandemic were conceived as advantageous to protect potentially vulnerable populations, such as older adults [29], from disease transmission; however, it has led to long-lasting effects on mental well-being.

Restricted LSM impedes older adults’ access to their [30-32] choice of environments and is associated with potential adverse mental health outcomes [27]. In addition, previous research has demonstrated that LSM restrictions reduce participation in out-of-home [33] activities and negatively affect QOL [9,34], which is in turn associated with loneliness [35].

Although pandemics create restricted mobility [36], older adults with knowledge of technology can capitalize on a digitally enabled AFE to avoid loneliness and LSM restrictions that create a strain on mental well-being. It enables older adults to engage in internet-based activities they may have enjoyed in person and gain access to health care, civic services, therapy, counseling [37], and resources that help with mental well-being [38,39]. We therefore expected both variables (LSM and loneliness) to impact AFEs negatively and for technology to moderate their impact.

Feldman and Oberlink [40] pioneered the concept of AFEs, identifying important components such as social engagement, enhancing independence, and optimizing mental functioning and well-being. Age-friendliness is derived from an ecological model of aging, in which a person’s mental well-being results from the interaction between their functional and cognitive capacity (competencies) and the environmental characteristics that exert pressure on these competencies (environmental stress) [41].

The age-friendly environment assessment tool (AFEAT) was designed to assess older adults’ perception of their environment and focuses on individual-oriented age-friendliness and individual-environment interaction, providing a more holistic picture [11,42,43]. Self-perceived AFEs are associated with improved QOL and mental health regardless of older adult frailty or abilities [8].

Although there is currently no universally accepted definition of an AFE, the World Health Organization (WHO) tentatively defines such communities as those where “policies, services, settings and structures support and enable people to age actively.” The WHO [1] publication imagines future cities to become more age-friendly to harness the latent potential of older adults through 8 intersecting domains addressing obstacles to older adults’ mental well-being: respect or social inclusion, outdoor spaces and buildings, housing, social participation, transportation, communication and information, civic participation and employment, and community support and health services. Explicit mention of technology is notably absent.

The WHO framework has been criticized for the exiguous technology element in the domains, prompting a reexamination [44]. Incorporating technology into AFEs in its broadest sense has become an increasingly important area for aging independence and mental well-being [45] in recent years.

Marston and van Hoof [44] discussed the incorporation and use of technology within the AFE assessment agenda, and a recent systematic umbrella review elaborated on the advantages of technology interventions for social connectedness [46]. Their call for the inclusion of digital technology as a domain in AFE evaluations is beginning to resonate.

For example, Pedell et al [47,48] advocated for digital elements to encompass all aspects of environmental age-friendliness, in addition to mental and physical aspects, to realize benefits for older adults. Moreover, Reuter et al [49] acknowledged the WHO’s age-friendly city initiative, considering an aging population amid increasing urbanization. However, they determined that such initiatives overlooked technology as a critical component of global digitalization.

### Research Objectives

Our study aimed to examine the potentially complex relationship between internal (loneliness) and external (LSM) factors that influence older people’s perception of AFEs and to determine whether technology moderates their impact. We aimed to answer a question with an important implication: should technology be included as a ninth domain in the WHO’s global age-friendly cities guide in the assessment of AFEs in cities and communities for older adult mental well-being?

We set out to test the following hypotheses:

- Hypothesis (H1): LSM restriction is negatively correlated with perception of AFEs.
- H2: Increased feelings of loneliness are correlated with poor perception of AFEs.
- H3: Technology use moderates the impact of LSM restrictions on the perception of AFEs.
- H4: Technology use moderates the impact of loneliness on the perception of AFEs after considering the LSM restriction effect.

## Methods

### Study Design

This was a cross-sectional quantitative observational design. This report follows the STROBE (Strengthening Reporting of Observational Studies in Epidemiology) guidelines [50] (STROBE checklist mentioned in Multimedia Appendix 1).

### Setting

Participants were recruited from the United Kingdom. Data were collected from January 16, 2020, to June 21, 2021, a period when social distancing mandates were enforced and social engagement outside the home was restricted.

### Participants

Eligible participants were required to be living in their own home, be proficient in English, and be 65 years or older. Older people who lived in nursing or care homes or those with cognitive decline or mental health issues were excluded. Volunteers were recruited via advertisements posted in resource centers for older adults, housing associations, third-sector organizations, social activity clubs, local senior groups, direct human interaction, and word-of-mouth recommendations. Volunteers were instructed to either call and leave a voicemail or send an email indicating their willingness to participate; a return call confirmed their eligibility.

G*Power software (Version 3.1; Erdfelder, Faul, and Buchner) was used to calculate the minimum sample size required for the empirical validation of the tested moderation model. Multiple regression was used, with effect size *f*^2^ of 0.15, power of 0.80, and 3 predictors. The recommended sample size was determined to be 87. A total of 110 participants enrolled; however, 18 did not complete all questionnaires and were excluded. The sample achieved included 92 people between the ages of 65 and 92 years (mean age 74.6, SD 7.23 years). All participants identified as either male or female, with more females (55/92, 60%) than males. More than 89% of the participants were White, with less than 11% of minority ethnicities (7 British Asian, 3 British Black). Having collated various demographic information such as age, gender, ethnicity, and education level, we were able to ascertain that participants emanated from diverse sociodemographic backgrounds.

### Variables and Measures

All participants filled out a health history questionnaire based on the Scientific Advisory Group for Emergencies (SAGE) Encyclopedia of Communication Research Methods [51].

Loneliness was measured using the 20-item UCLA (University of California, Los Angeles) Loneliness Scale [52], with scores ranging from 20 to 80. Higher scores reflected higher loneliness (Cronbach α=0.88).

Utilization of technology was evaluated using the Technology Experience Questionnaire [53]. The participants were given a list of technologies (communication, computer, daily, health, recreational, and transportation technology) and asked to rate their familiarity with and use of each on a 5-point scale. Scores ranged from 0 to 180, with higher scores indicating greater use and familiarity with technology (Cronbach α=0.84).

The perception of the AFE was assessed by the AFEAT. This is a 10-item measure that uses a 5-point Likert scale, scoring items from (1) strongly disagree to (5) strongly agree and gauging participants’ perceptions of their home, their local communities, the resources within the environment, and their appropriateness for meeting their daily needs. The scores ranged from 0 to 50, with higher scores representing a more positive perception of the age-friendliness of the environment (Cronbach α=0.75).

LSM was measured using the Life-Space Questionnaire [26]. Participants were asked yes or no questions about specific places they visited in the last 4 weeks, starting with another room in their current residence and increasing the distance to a location outside England. The scores ranged from 9 to 18, with higher scores demonstrating greater restriction of LSM (Cronbach α=0.90).

### Procedure

Telephone surveys collected information on loneliness, technology use, LSM, and perceptions of AFEs, in addition to basic demographic information (eg, age, education, gender, and ethnicity). Google Analytics was used to collect and tabulate the data. Participants completed the assessments across 14 months.

### Statistical Analysis

Analyses were performed using IBM SPSS version 28, using a minimum significance level of 95% probability. There were no missing data, and participants completed all questions. The variables of AFEAT, loneliness, technology use, and LSM were inspected for kurtosis and skewness to assess their distribution deviations from normality via a histogram with simulated overlapping normal curves. Moreover, the homoscedasticity of the residuals was checked using a standardized residual versus a standardized predicted plot. Using the Mahalanobis (*P*<.001) and Cook distances, we determined whether high leverage points, significant outliers, or highly influential points exist by examining a scatterplot matrix of the dependent and continuous independent variables. A linear regression was performed to check the included variance caused by the data point and if it needed to be removed from the dataset. The criterion for discarding observations was the inability to meet 2 of the distance measures’ 3 gauges. However, no outliers were found that would significantly impact the findings, and thus, none were removed. The confirmation of the independence of the observations and the assumption of no autocorrelation in the residuals was checked using the Durbin-Watson *d*-statistic.

The initial descriptive analyses contained means, frequencies, and SD. Pearson product-moment correlation coefficients (*r*) were calculated to determine whether associations exist between the variables. The same correlational analysis was used to determine whether the perception of AFE is correlated with LSM during the pandemic (H1) and whether loneliness is correlated with the perception of AFEs (H2).

Hayes’ [54] PROCESS macro for SPSS with model 1 was applied to investigate the moderating effects of technology use on the relationship between LSM and AFE (H3) and technology use on the relationship between loneliness and AFEs. If the standardized coefficients of the interaction terms were significant (*P*<.05) or marginally significant (*P*<.09), we conducted a simple slope test to examine the interaction effect at different levels to reveal the nature of significant interactions to further explain the moderating effect.

### Ethical Considerations

Participants accessed an information sheet either via email or read on the phone and were allowed to ask questions before giving their consent. Before completing the questionnaires, each participant gave informed consent to volunteer without compensation and participate in the study. All participants were fully anonymized.

All participants were informed of their rights to withdraw at any point in the research and informed about anonymity. The ethical procedures were aligned with the guidelines of the British Psychological Society, and the study received ethical approval from the Lancaster University Faculty of Health and Research Ethics Committee (reference number FHMREC19121). Data captured via telephone first confirmed the participant’s identity and was recorded in spreadsheets and anonymized thereafter.

## Results

### Overview

Table 1 shows mean, SD, kurtosis, and skewness values and Shapiro Wilk test results. Kurtosis and skewness values had a relatively small range of ±1; we determined that the normal distribution deviation was insignificant. The distributions of the variables of loneliness, AFE, technology experience, and LSM were close to normal.

**Table 1:** Descriptive statistics for loneliness, life-space, age-friendly environment assessment tool, and technology experience (N=92).

	Scores						
	Minimum	Maximum	Mean (SD)	Skewness	Kurtosis	Shapiro Wilk test	
UCLA^a^ loneliness score	21	80	47.49 (17.814)	0.204	−1.630	0.192^b^	
Life-space mobility	10	18	13.70 (1.595)	0.515	0.035	0.201^b^	
Age-friendliness of environment	0	35	19.51 (9.687)	0.019	−1.399	0.168^b^	
Technology experience	48	175	116.87 (40.951)	−0.260	−1.624	0.204^b^	

^a^UCLA: University of California, Los Angeles.

^b^*P*<.001 under moderate.

Participants demonstrated high levels of loneliness, with 44% of older adults demonstrating loneliness scores greater than 50, with scores above 40-50 considered moderate loneliness and scores greater than 50 considered high [55]. LSM scores were high, with more than 93% of the participants scoring >11, showing that they had not been outside their immediate town. Previous prepandemic studies with similar sample sizes and methodology reported almost half that score [26]. The perceptions of AFEs were mixed, with a mean score of 19.51 (SD 9.69), demonstrating both positive and negative perceptions.

Prepandemic data from Garner and Holland’s [8,56] studies provided a mean of 42.2, taken from 132 participants based in England, indicating a more positive perception of AFEs before the pandemic. Most participants scored above 125 (56%) for technology, demonstrating frequent use and familiarity with technology in general [53].

Next, we calculated the Pearson correlation coefficients to establish the relationships between loneliness, technology, LSM, and AFE perception to test H1 and H2. A correlation matrix of the variables was examined and is presented in Table 2.

**Table 2:** Correlational analysis between variables (N=92).

Variables		UCLA^a^ Loneliness Scale	Life-space mobility	Age-friendliness of environment	Technology experience
**UCLA Loneliness Scale**					
	*r*	1	−0.483^b^	−0.698^b^	−0.631^b^
	*P* value	—	.002	.006	.003
**Life-space mobility**					
	*r*	−0.483^b^	1	0.461^b^	0.430^b^
	*P* value	.003	—	.003	.004
**Age-friendliness of environment**					
	*r*	−0.698^b^	0.461^b^	1	0.667^b^
	*P* value	.003	.004	—	.003
**Technology experience**					
	*r*	−0.631^b^	0.430^b^	0.667^b^	1
	*P* value	.003	.004	.002	—

^a^UCLA: University of California Los Angeles.

^b^*P*<.01.

^c^Not applicable.

### LSM is Negatively Correlated With the Perception of AFE During the Pandemic (H1)

The correlation between LSM and AFE perception was statistically significant (*r*=0.461, *P*<.001) but positively correlated, which was contrary to the hypothesis (Table 2). This meant that higher LSM scores associated with restricted mobility were correlated with a greater positive perception of AFEs. Although this rejected H1, it was a notable result.

### Loneliness Is Negatively Correlated to a Perception of AFEs (H2)

The correlation between loneliness and AFE perception was statistically significant (*r*=−0.698, *P*<.001) and negatively correlated. This meant that greater loneliness was correlated with more negative perceptions of AFEs, thus confirming H2.

### Technology Use Moderates the Impact of LSM Restriction on the Perception of AFEs (H3)

**Figure 1:**
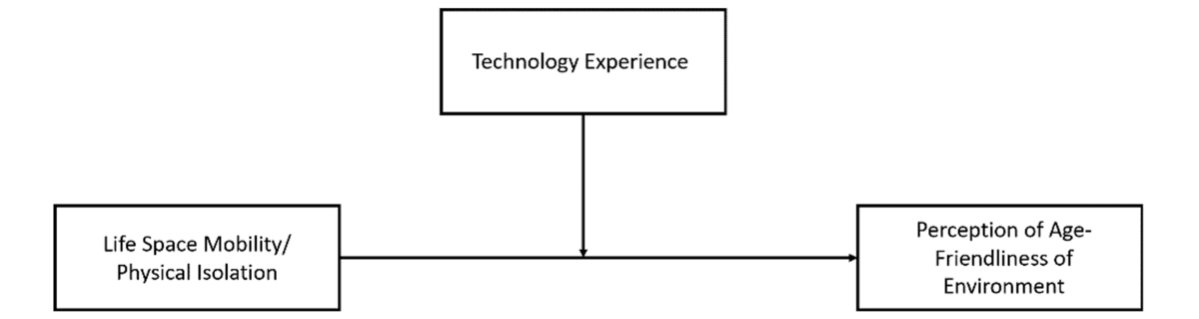
Moderating role of technology experience on the relationship between life-space mobility and age-friendliness of environments.

Model 1 was used in the PROCESS 4.0 macro for SPSS to examine the moderation effect proposed in H3 [54], as shown in Figure 1, which shows the moderation role played by technology use in the relationship between LSM and AFE perception.

Here, all continuous variables were converted to *z* scores for use in the model as suggested by Frazier et al [57] and Hayes [54] (ie, via *z* scoring, expressed as the deviation from their sample means in SD units). The unconditional interaction of LSM and technology use was insignificant (β=0.1921, *t*_1_=1.963; *P*=.06; Table 3).

All variables in the model are standardized and brought into the regression equation.

**Figure 2:**
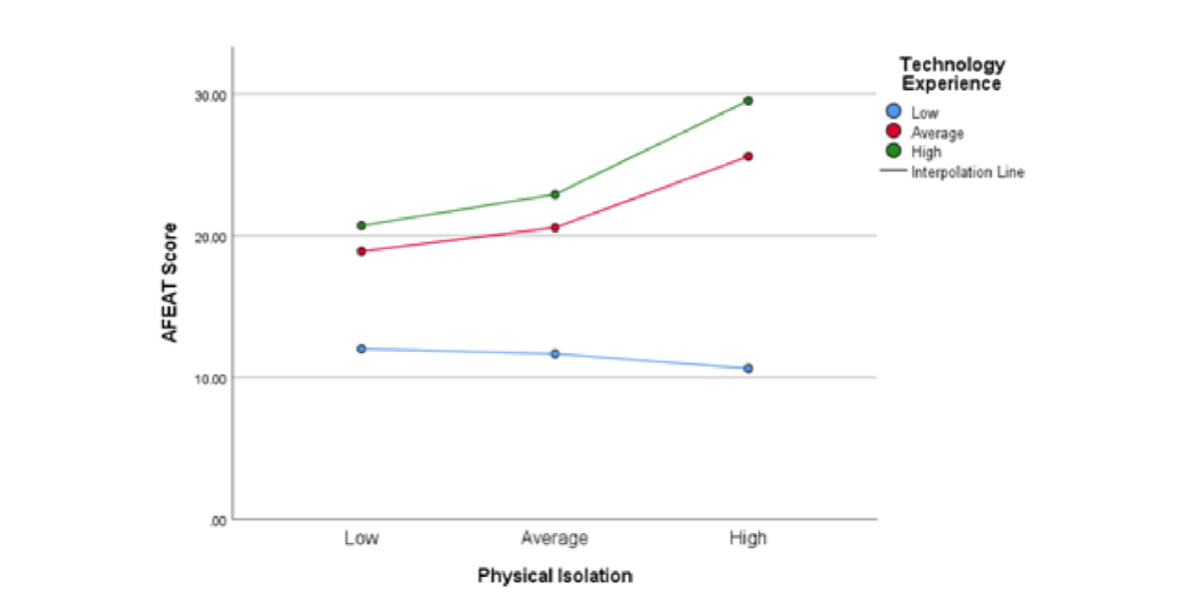
Moderating role of technology experience on the relationship between life-space mobility and age-friendly environment (simple slope test). AFEAT: age-friendly environment assessment tool.

Using the Aiken and West [58] method, a simple slope test was used to analyze the conditional effect of technology use between LSM and AFEs (ie, whether technology use moderates the relationship between LSM and AFEs). As illustrated in Figure 2, when technology experience was high, LSM and AFEs were significantly positively correlated (β simple: mean 1, SD 2.2037, *t*=3.2216; *P*=.003), indicating that older adults’ perception of their environment was more positive when they used technology more.

In contrast, the relationship between LSM and AFEs was not obvious when the technology experience was low (β simple: mean −1, SD −0.3429, *t*=−0.3510; *P*=.73). Thus, there appears to be a positive relationship between LSM restriction and perception of AFEs when technology use was high but not when it was low (explaining the unexpected direction of H1). Furthermore, note the slight downward slope indicating the negative impact of LSM restrictions on the perception of AFEs when technology experience was low. This hints that in the absence of technology use, LSM restrictions had a detrimental impact on the perception of AFEs. This confirms H3 and explains the initial rejection of the unconditional (overall) moderation impact of technology experience on LSM by a small margin (*P*=.06).

**Table 3:** Interaction between life-space mobility and technology experience.

			Overall fit indicators							Significance of standardized coefficient		
			*R*		*R* ^*2*^		*F* test (*df*)		β			*t* test (*df*)
**Variables**			0.71		0.50		29.7962 (1,90)					
	Life-space mobility (ZLS1)							0.1787			2.1002^a^ (90)	
	Technology experience (ZTE1)							0.6155			7.1839^a^ (90)	
	ZLS1*ZTE1							0.1921			1.9628 (90)	

^a^*P*<.001.

### Technology Use Moderates the Impact of Loneliness on the Perception of AFEs After Considering the Impact of LSM (H4)

**Figure 3:**
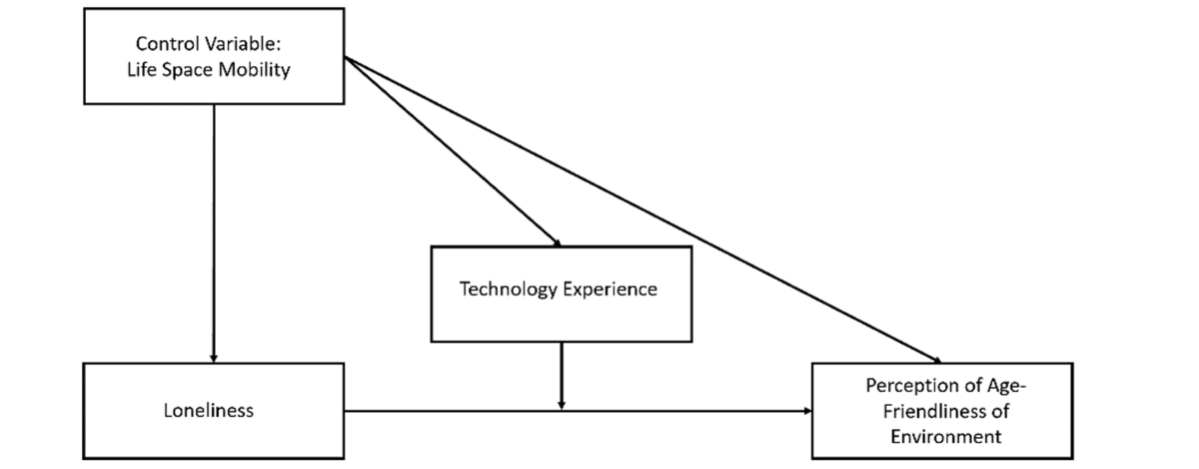
Moderating effect of technology experience in the relationship between loneliness and age-friendliness of environments after controlling for life-space mobility.

Model 1 was used in the PROCESS 4.0 macro for SPSS to examine the moderation effect of technology use on the relationship between loneliness and perception of AFEs, as proposed in H4 [54] and shown in Figure 3, which shows the moderating effect of technology experience on the relationship between loneliness and the perception of AFEs while controlling for LSM.

All continuous variables were converted to *z* scores. As shown in Table 4, the relationship between loneliness and technology experience was significant (β=−0.3829, t_90_=−5.1518; *P*<.001), but the impact of LSM when added to the regression model was not significant (β=0.7151, t_90_=1.665; *P*=.07), showing that although technology use had a moderating impact on the relationship between loneliness and AFE perception, the contribution of LSM was not significant on the model once these other variables had been taken into account.

All variables in the model are standardized and brought into the regression equation.

**Figure 4:**
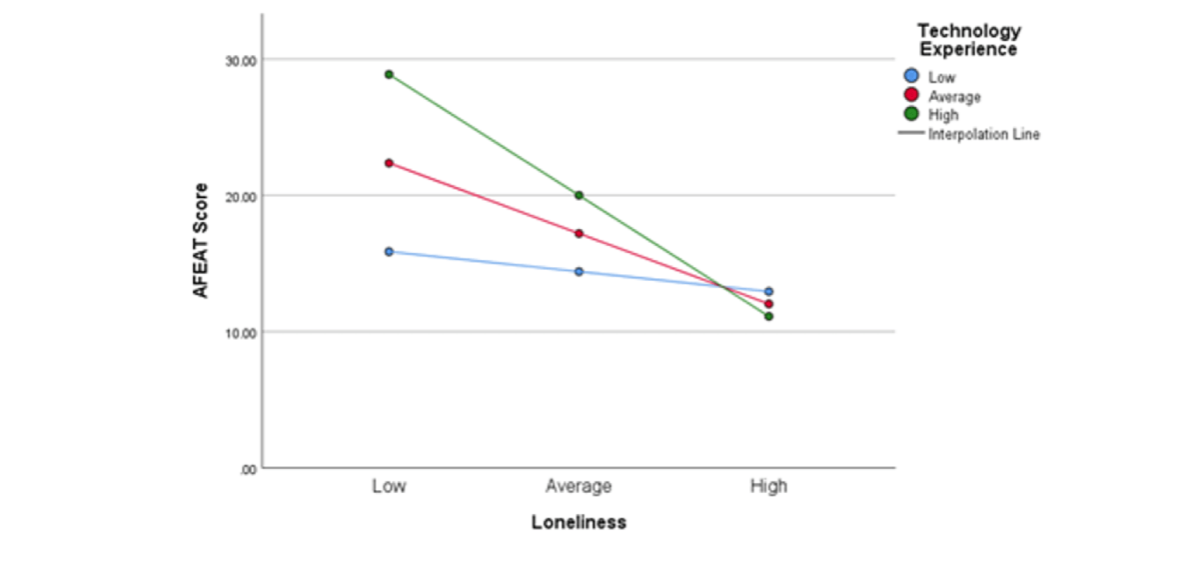
Moderating effect of technology experience in the relationship between loneliness and age-friendliness of environments after controlling for life-space mobility (simple slope test).

We then used the simple slope test to analyze the conditional effect of technology on the impact of loneliness on the perception of AFEs to further understand the impact. As evidenced in Figure 4, the link between loneliness and AFEs was not as obvious when technology was low (β simple: mean −1, SD −0.0819, *t*_1_=−1.5394; *P*=.13). Conversely, when technology was high, the impact of moderation was apparent more clearly (β simple: mean 1, SD −0.4983, t_90_=−7.2636; *P*<.001). Thus, we can conclude that technology moderates the impact of loneliness on the perception of AFEs when the technology experience is high, confirming H4.

**Table 4:** The relationship between loneliness and technology experience and the impact of life-space mobility.

		Overall fit indicators				Significance of standardized coefficient	
		*R*	*R* ^*2*^	*F* test (*df*)	β		*t* test
**Variables**		0.82	0.68	45.8722 (1,90)			
	Loneliness (ZL1)	—^a^	—	—	−0.5335		−6.2794^b^
	Technology experience (ZTE1)	—	—	34.78 (1,90)	0.2888		3.5746^b^
	ZL1*ZTE1	—	—	—	−0.3829		−5.1518^b^
	Life-space mobility (ZLS1)	—	—	—	0.1177		1.665

^a^Not applicable.

^b^*P*<.001.

## Discussion

### Principal Findings

This study found higher levels of loneliness during the height of the COVID-19 pandemic among the older adult sample compared with the commonly available prepandemic data. For instance, Victor and Bowling [59] reported an average loneliness level of 30%, whereas Hawkley and Cacioppo [60] found the prevalence of loneliness in older adults to be approximately 25%, compared with the 44% found in this study. Numerous studies have found an increase in loneliness during the pandemic [61-63].

Higher loneliness was found to be correlated with a more negative perception of AFE, confirming H2 and in line with what has been theorized by the prevailing literature [18-20,22]. Shortfalls in emotional and social fulfillment have been highlighted as a predictor of loneliness, and the lack of opportunities for older adults to socialize, access important key services, and ability to engage with the community may have worsened perceptions. When older adults perceive that their desired quantity or quality of social engagements is met, they are less likely to experience loneliness and may also have favorable attitudes toward AFEs. Previous research has suggested that this relationship is likely to be complex [19] and bidirectional. AFE perception can be an indicator of a mechanism for aging-in-place, preserving older adults’ physical setting of choice, providing a sense of community attachment, and allowing them to engage with a developed social network, with associated familiarity and better mental well-being.

This study found that greater use of technology clearly moderated the relationship between loneliness and the perception of AFEs. It also moderated the LSM-AFE relationship. Furthermore, the ability to use technology successfully to adapt to challenging experiences during lockdown emerged as a potential buffer against the impact of loneliness on AFE perception. Other studies that examined technology use during the pandemic revealed an increase, indicating adaptation to LSM restrictions via alternative pathways [37,64]. An increase in LSM restriction was linked to a positive perception of AFEs, rejecting H1, which at first seemed counterintuitive. However, the moderation effect of technology, such that this relationship existed only when technology use was high, explained the unexpected direction of the relationship. Older adults were overcoming physical restrictions barriers, where the ability to replace previously in-person activities with those online may have impacted perceptions. Other studies that examined AFEs also noted an increase in positive perception of AFEs as the pandemic progressed, but these could also have been linked to the easing of restrictions [56]. Overall, technology use may have improved the negative impacts of loneliness on the perception of AFEs helping with older adult mental health.

In testing H4, we determined that when technology experience was high, it had a moderation effect on the loneliness-AFE relationship. Ng et al [16] attempted to explain the relationship between internet use and loneliness through a moderation-mediation mechanism between internet use, perception of AFE, and loneliness, and depression. Their findings were consistent with those of Park et al [24] and Domènech-Abella et al [65], who confirmed the moderating effect of the internet on the age-friendliness-loneliness-depression mechanism, which may also explain our results. For example, Booth et al [66] discovered a partial mediation effect of feelings of helplessness, social isolation, depression, and distrust between psychological distress and perceived AFE (especially concerning security). Social isolation is a well-established predictor of loneliness and depression, and loneliness has also been linked in the literature. Taken together, these hint at a causal route between loneliness and perception of AFEs. Increased anxiety can lead to increased loneliness and mental health issues, potentially leading to a reduced fit between the older adult and the environment [67,68].

Despite studies highlighting the potential moderating role of technology between loneliness and AFE perception, previous results were always unclear about a direct link between these 3 variables. Our study found a clear moderating role for technology in the relationship between loneliness and AFEs, which is a notable finding.

When using the simple slope test, where technology use was low, we found that LSM negatively affected perceived AFEs when technology use was low. This explained our initial counterintuitive result and supported earlier findings [24]. To substitute or overcome confinement, older adults may have developed alternative routes of access to AFE domains, such as social, civic engagement, and access to services through technology.

Access to the internet may have helped reduce older people’s boredom, and there have been examples from recent studies where older adults were able to access informal help networks, reading, or online game groups, as well as participate in community-based activities like attending virtual church gatherings [69,70]. Alternative social and emotional outlets to combat loneliness through access via the internet, to previously in-person services (eg, primary care, counseling) may have contributed to network socialization, allowing older adults to continue feeling like they are a part of their environment and reducing loneliness.

The implications of our study can be applied to situations outside of the pandemic context. Studies have highlighted that older adults prefer to live in their own homes and interact with their local community, where they have developed relationships over time and do so as long as possible [71,72]. A negative perception of AFEs can be viewed as a barometer indicating poor person-environment fit [72], associated with poorer mental health outcomes [25,71]. Therefore, technology could be a solution for those who ordinarily have limited LSM, are at risk for social isolation, and have a negative perception of AFEs.

Our study confirmed the link between LSM, loneliness, and perception of AFEs, as well as the moderating role technology played during the COVID-19 pandemic, advancing the findings of previous studies in this rapidly evolving body of literature. We also strengthened the argument that the WHO’s Global Age-Friendly Cities framework would benefit older adults more by including technology use as an additional ninth domain.

### Study Limitations

This study had several limitations. For example, the sampling could have been predisposed to participants literate in digital resources and more socially connected. Generally, such participants may have experienced less loneliness [73]. Furthermore, a cross-sectional design cannot establish causality [74]. Although the sample size was small, the statistical power, effect size, precision, type, complexity of analysis, study population variability, and homogeneity overcame this shortcoming. The results are an important contribution to the discourse on the role being played by AFEs in the mental well-being of older adults and the role of technology.

Although it is unlikely that participants will experience the same level of LSM restrictions after the pandemic, this allowed us to examine the studied measures in a normally inaccessible environment. However, we cannot conclude with certainty that the pandemic caused the observations because we did not have a prepandemic assessment for the same participants. Nevertheless, other prepandemic studies supported our hypothesis of mobility restrictions’ impact on loneliness.

### Conclusion

Despite the limitations, the results suggest that the vulnerability of older adults during the pandemic and their exposure to loneliness and negative perceptions of AFEs increased, and technology played an important role in moderating these influences. Given the advancements in technology, the WHO’s 8 domains of AFEs may be obsolete with their narrow implicit recognition of technology. Our study demonstrates the significance of an explicit recognition of technology in the evaluation of AFEs as an integral component of all aspects of older adults’ daily lives. Future studies may also wish to look further into the impact of demographics and differences between genders.

Community and mental health service access could be improved by providing online access. Older adults need cheap access to internet infrastructure, and health community centers should provide technology training, attend online meetings, or use mental health applications.

Researchers in the field of loneliness in older adults are encouraged to use our results to inform initiatives to reduce the mental health risks for older adults in vulnerable crises, such as the pandemic and civil insurrections [75,76]. Appropriate consideration of these factors will aid decision makers in developing robust and effective strategies during times of crisis, as well as in assisting an aging population with aging-in-place.

## Appendix

Multimedia Appendix 1 STROBE Checklist.

## Abbreviations

AFE: age-friendly environment
AFEAT: Age-Friendly Environment Assessment Tool
H: hypothesis
LSM: life-space mobility
QOL: quality of life
SAGE: Scientific Advisory Group for Emergencies
STROBE: Strengthening Reporting of Observational Studies in Epidemiology
WHO: World Health Organization
